# Design, synthesis and anticancer activities evaluation of novel pyrazole modified catalpol derivatives

**DOI:** 10.1038/s41598-023-33403-9

**Published:** 2023-05-12

**Authors:** Yuanfang Kong, Shuanglin Liu, Shaopei Wang, Bin Yang, Wei He, Hehe Li, Siqi Yang, Guoqing Wang, Chunhong Dong

**Affiliations:** 1 Henan University of Chinese Medicine, Zhengzhou, 450046 Henan China; 2 Henan Polysaccharide Research Center, Zhengzhou, 450046 Henan China; 3 Henan Key Laboratory of Chinese Medicine for Polysaccharides and Drugs Research, Zhengzhou, 450046 Henan China; 4 Department of Applied Chemistry, Zhengzhou University of Light Industry, ZhengzhouHenan, 450001 China

**Keywords:** Chemical modification, Drug screening, Medicinal chemistry

## Abstract

Catalpol, a natural product mainly existed in plenty of Chinese traditional medicines, is an iridoid compound with the comprehensive effects on neuroprotective, anti-inflammatory, choleretic, hypoglycemic and anticancer. However, there are some disadvantages for catalpol such as a short half-life in vivo, low druggability, stingy binding efficiency to target proteins and so on. It is necessary to make structural modification and optimization which enhance its performance on disease treatments and clinic applications. Pyrazole compounds have been reported to have excellent anticancer activities. Based on the previous research foundation of our research group on iridoids and the anticancer activities of catalpol and pyrazole, a series of pyrazole modified catalpol compounds were synthesized by principle of drug combination for serving as potential cancer inhibitors. These derivatives are characterized by ^1^H NMR, ^13^C NMR and HRMS. The efficacy of anti-esophageal cancer and anti-pancreatic cancer activities were evaluated by the MTT assay on two esophageal cancer cells Eca-109 and EC-9706, and two pancreatic cancer cells PANC-1, BxPC-3 and normal pancreatic cell line HPDE6-C7, which showed that the compound **3e** had strong inhibitory activity against esophageal cancer cells, this providing a theoretical basis for the discovery of catalpol-containing drugs.

## Introduction

Cancer is a life-threatening disease and remains a major health problem around the globe, which is the second most prevalent disease after cardiovascular disease^1^. Esophageal cancer^2–4^ and pancreatic cancer^5^ are the most common cancers in digestive and gastrointestinal tract. Conventional chemotherapy drugs play a role through cell toxicity. Due to the inability to accurately identify cancer cells and play a wide range of cytotoxic effects, some patients show obvious toxic and side effects with the prolongation of medication time, and some even produce drug resistance. These toxic and side effects limit the treatment of cancer. Therefore, the development of safe, low-toxic and efficient anticancer drugs has become the focus of drug researchers.

**Figure 1:**
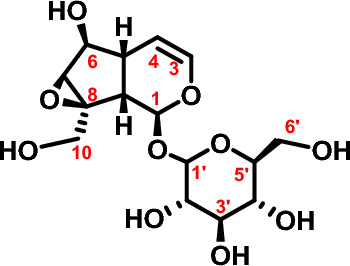
Chemical structure of catalpol.

It is an essential compound library for the development of various new structural drugs from Chinese medicine. Catalpol is an important active ingredient of Chinese medicine, which has significant inhibitory effects on breast cancer^6^, gastric cancer^7^, lung cancer^8^ and colorectal cancer^9^. Due to the complexity of catalpol molecular structure, low activity intensity and poor drug-forming ability, it is necessary to modify the structure of catalpol^10^. The chemical structure of catalpol was shown in Fig. 1.

**Figure 2:**
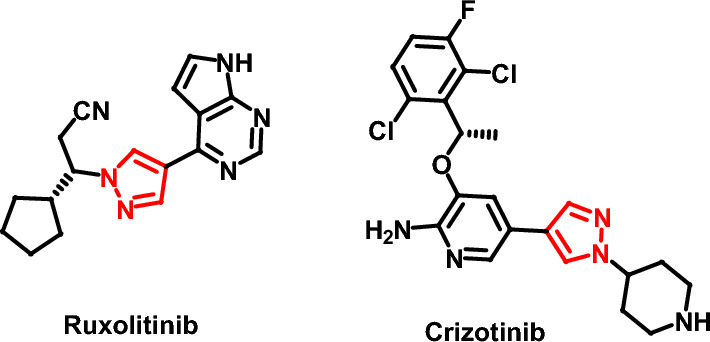
Pyrazole based anticancer drugs.

Numerous heterocyclic compounds have attracted the medicinal chemists′attention due to their anticancer activities, and the introduction of heterocyclic groups in various natural products can enhance their biological activity^11–14^. Pyrazole motif received an extensive attention due to its varied pharmacological utilities^15,16^, it emerges as a useful pharmacophore scaffold in the synthesis of potent anticancer agents^17^. For example, Ruxolitinib and Crizotinib are two examples of pyrazole based anticancer drugs which are currently available in the market (Fig. 2) ^18^.

**Figure 3:**
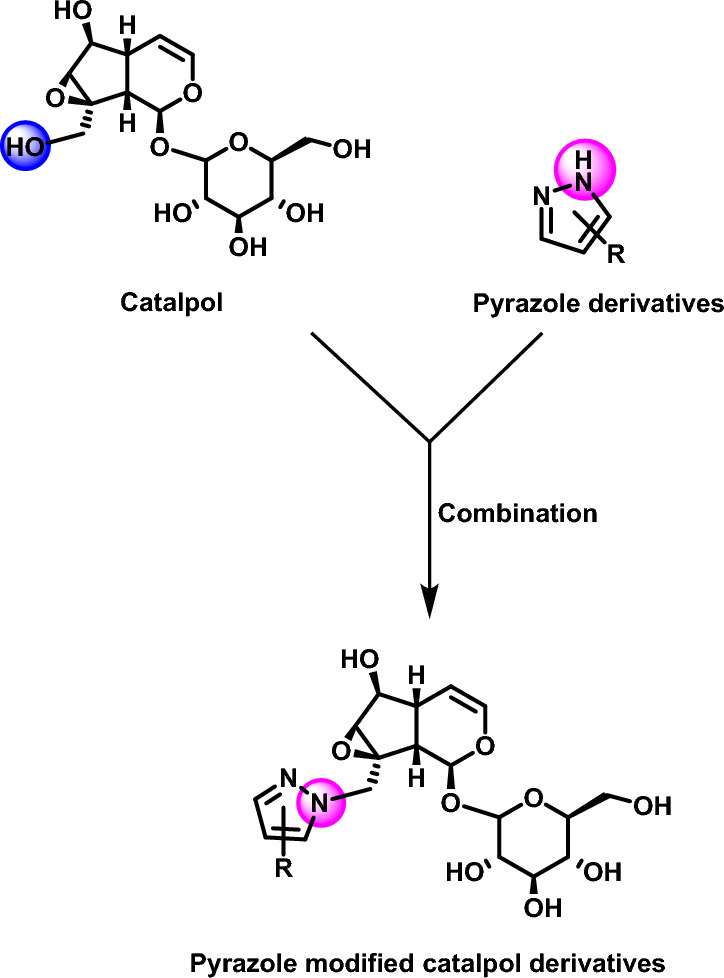
Design of pyrazole modified catalpol derivatives.

Based on the previous research foundation of our research group on iridoids^19,20^ and the anticancer activity of catalpol and pyrazole, a series of pyrazole modified catalpol compounds were synthesized by the principle of drug combination (Fig. 3). Different substituents were introduced on the pyrazole group in order to obtain catalpol derivatives with anticancer activities.

## Results and discussion

### Chemistry

Catalpol is a polyhydroxyl compound, in which the reactivity of hydroxyl groups at different positions varied, thus, it is difficult to selectively modify specific hydroxyl groups at the particular position. In the design of the synthetic route of the target compound, the multi-functional characteristics of catalpol should be considered and appropriate reaction conditions should be selected in every step of the synthesis to avoid damaging the parent structure of catalpol. In the structure of catalpol, the glycosidic bond was easily destroyed by enzymatic hydrolysis, acid hydrolysis and alkaline degradation, and epoxides also can undergo ring-opening reactions with acid and strong bases. Furthermore, the functional group of cycloalkene ether bond in catalpol is extremely active, and it is prone to addition reaction, oxidation reaction, polymerization reaction, and the addition reaction of carbon–carbon double bonds and halogens can also occur under acidic conditions.

**Figure 4:**
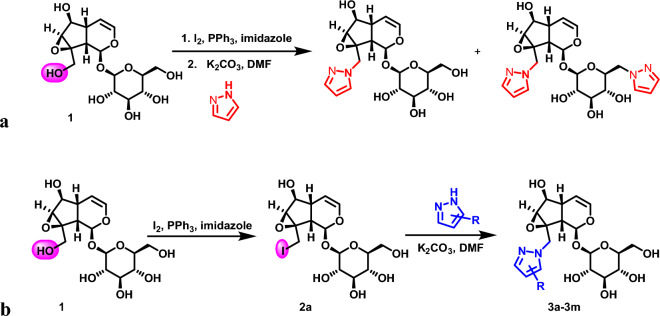
Synthesis of pyrazole modified catalpol derivatives. (**a**) One-pot synthesis of pyrazole modified catalpol derivatives. (**b**) Stepwise synthesis of pyrazole modified catalpol derivatives.

The experimental result showed various reaction products were generated from “one-pot” synthesis, which led to tremendous difficulty in separation and purification afterward (Fig. 4a). Therefore, the multistep synthesis approach would be a priority consideration for this experimental design. Here, a series of pyrazole modifed catalpol derivatives were synthesized by replacing C10-position hydroxyl groups of catalpol with the pyrazole (Fig. 4b).

**Figure 5:**
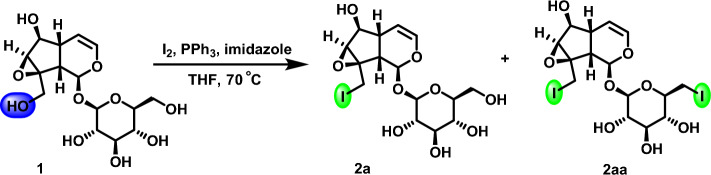
Synthesis method of catalpol hydroxyl iodide.

The reaction method of hydroxy halogenation on catalpol and redirected introduction of heterocyclic pharmacophore was explored. Imidazole, triphenylphosphine and iodine were selected to iodinate catalpol hydroxyl selectively. The reaction solution is refluxed at 70 °C to obtain new products **2a** and** 2aa**. In order to obtain compound **2a** selectively, the reaction conditions of catalpol hydroxyl iodination were screened (Fig. 5). In this reaction, the hydroxyl group in catalpol was poorly selected to generate two iodine products. In order to obtain a single product, the reaction conditions must be optimized.

In the reaction, since the structure of catalpol contained two primary hydroxyl groups, it is difficult to obtain a single product in the experiment. Through molecular simulation docking, there is little difference in activity between the two primary hydroxyl groups. However, the temperature, the initial concentration of iodine impinges heavily on the product category, yield and diversity. According to the reported reaction mechanism, it is necessary to explore the reaction equivalence ratio, temperature, initial concentration of iodine, and solvent conditions.

According to the result of catalpol iodization reaction, shown in Table 1, the selectivity of iodine reaction was greatly affected by reaction temperature. The higher reaction temperature, the more diversified products are generated. When 1.5 eq and 3 eq of iodine are respectively dissolved in ultra-dry tetrahydrofuran refluxing at 70 °C, the selectivity is seemingly pretty poor. When 1.5 eq of iodine is used for the reaction at 70 °C, the catalpol keep unreacted. When 3 eq of iodine is used for the reaction at 70 °C, the reaction is completed in 0.5 h, so the reaction was affected by the initial concentration of iodine as raw material (Entries 1–2, Table 1). Considering that the selectivity of the reaction was affected not only by the reaction temperature, but also by the initial concentration of the raw material iodine, the reaction selectivity was increased by lowering the reaction temperature under 3 eq of iodine at room temperature. It is found that the reaction was completed in 1 h, but the reaction selectivity was still poor, and the yield of compound **2a** was low; when the reaction was carried out with 3 eq of iodine at 0 °C, the reaction selectivity was relatively high with almost only the product **2a**, although the catalpol still remained after prolonged time of reaction. When the temperature of the reaction system was increased or iodine as the feedstock was added, there was surplus of feedstock catalpol (entries 3–4, Table 1). The reactions were explored by different initial concentrations of iodine at 0 °C (entries 5–7, Table 1). The experimental results showed the maximum yield of compound **2a** was 70% when the amount of iodine was 6 eq and the reaction time was 18 h. When tetrahydrofuran (THF) was used as the reaction solvent, the synthesis process was not a homogeneous reaction system and there is solid precipitation in the process of reaction, so *N*, *N*-dimethylformamide (DMF) was used as the additional reaction solvent (entries 8–10, Table 1). Meanwhile, iodine with different initial concentrations were selected for the reaction, and the white solid (Ph_3_PO) was found to be the by-product. The reason for the low reaction yield is that the reaction solvent DMF was used to dissolve the solid in the experiment, which slows down the reaction speed and affects the output of the product. The reactions were also explored by different initial concentrations of iodine at − 10 °C and − 20 °C (entries 11–13, Table 1). The results showed that the raw material catalpol was not completely reacted at each reaction temperature, and the reaction selectivity was the same as that at 0 °C.

**Table 1:** Exploration of the reaction conditions of catalpol hydroxyl iodination.


Entry	Sol	Add Sol.^(b)^	I_2_, PPh_3_ (eq)	Temp. (^o^C)	Time (h)	Yield (%)
1^(a)^	THF	–	1.5	70	–	–
2	THF	–	3	70	0.5	34
3	THF	–	3	rt	1	40
4^(a)^	THF	–	3	0	–	–
5^(a)^	THF	–	4	0	–	–
6	THF	–	5	0	20	59
7	**THF**	–	6	0	18	70
8^(a)^	THF	DMF	3	0	–	–
9^(a)^	THF	DMF	4	0	–	–
10	THF	DMF	6	0	6	45
11^(a)^	THF	DMF	5	− 10	–	–
12^(a)^	THF	DMF	6	− 10	–	–
13^(a)^	THF	DMF	10	− 20	–	–

^(a)^The raw material catalpol has not reacted completely ^(b)^DMF is the second reaction solvent, and the ratio with THF is 1:4.

Through the exploration of reaction conditions of catalpol iodination, the optimal reaction conditions were finally determined as the ratio of *n*(catalpol):*n*(iodine):*n*(triphenylphosphine):*n*(imidazole) was 1:6:6:12, the solvent was ultra-dry tetrahydrofuran, the reaction temperature was 0 °C.

**Figure 6:**
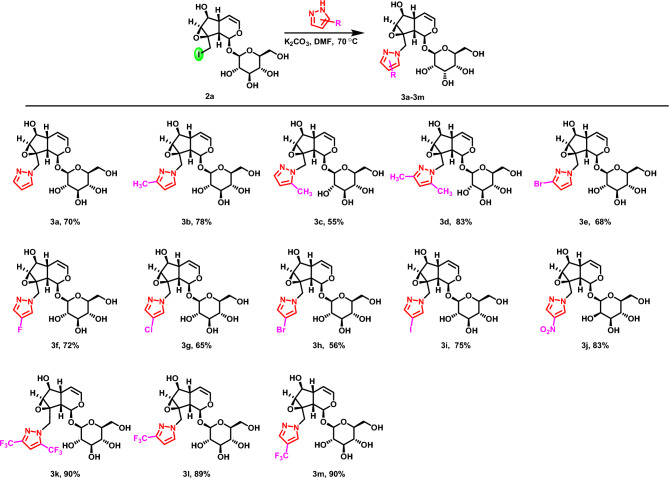
Synthesis of pyrazole modified catalpol derivatives.

A series of C10-position pyrazole modified catalpol derivatives (**3a–3m**) were synthesized by C10-iodocatalpol treated with different substituted pyrazole derivatives under K_2_CO_3_ in DMF at 70 °C (Fig. 6). When the unsubstituted pyrazole was used, the reaction could react and obtain the yield of 70% (**3a**). When pyrazole was substituted by the electron-donating group (–CH_3_), the reaction can proceed well, whether in the C3- or C5-substituted (**3b–3c**) or C3- and C5-substituted pyrazoles (**3d**). Pyrazole modified catalpol derivatives can also be obtained in moderate yields when C4-position pyrazole was substituted by halogen atom (**3e–3i**). When the C4-position pyrazole was replaced by electron-withdrawing groups (–NO_2_, –CF_3_), this reaction can be obtained in high yields (**3j–3m**). Under this optimal reaction condition, both electron-donating and electron-withdrawing substituted pyrazole compounds can react with catalpol to produce a series of high-yield pyrazole modified catalpol derivatives.

### Biological

The target compounds were evaluated for their anticancer activity against human cancer cell lines of different origins, including Eca109 (human esophageal cancer cell line) and EC9706 (human esophageal cancer cell line), PANC-1 (human Pancreatic cancer cell line), BxPC-3 (human pancreatic cancer cell line) and HPDE6-C7 (human normal pancreatic ductal epithelial cell line) via MTT assay.

Human pancreatic cancer cells BxPC-3 and PANC-1 and human normal pancreatic ductal epithelial cell HPDE6-C7 were purchased from the Cell Bank of Shanghai Institute of Biochemistry, Chinese Academy of Sciences. Human esophageal cancer cell line Eca109 and EC9706 used in the experiments were obtained from the American Type Culture Collection (ATCC, Manassas, VA).

#### In vitro esophageal cancer cells inhibitory activities

MTT method was used to investigate the inhibitory effect of the target compound on the cell formation of Eca109 and EC9706 cells cultured in vitro. The drug concentration was 2 mM. From the preliminary results in Table 2. It can be seen that catalpol has weak cell inhibition effect (Supplementary Fig. S3). And compound **3e** has a strong inhibitory effect on Eca109 and EC9706 cells. For Eca109 cell, the cell survival rate was 62% at 24 h and 35% at 48 h after treatment with 2 mM of drug concentration. For EC9706 cell, the cell survival rate was 48% at 24 h and 31% at 48 h after treatment with 2 mM drug concentration (Supplementary Fig. S4).

**Table 2:** In vitro esophageal cancer cells viability of pyrazole modified catalpol derivatives.

Esophageal cancer cells viability (% of control)				
Number of compounds	Eca109		EC9706	
	24 h	48 h	24 h	48 h
**1**	82.9 ± 5.500	71.100 ± 4.900	83.000 ± 3.300	85.700 ± 6.300
**3a**	–	–	–	–
**3b**	–	–	88.900 ± 24.600	84.500 ± 14.400
**3c**^**(a)**^	75.800 ± 8.600	78.200 ± 8.900	64.200 ± 9.600	89.200 ± 10.500
**3d**^**(a)**^	84.800 ± 9.100	83.300 ± 23.100	77.600 ± 11.300	86.400 ± 5.000
**3e**	62.900 ± 3.000	36.100 ± 3.400	48.600 ± 3.800	31.900 ± 0.300
**3f**	74.400 ± 1.000	86.000 ± 1.700	81.100 ± 3.000	80.800 ± 2.100
**3g**^**(a)**^	98.500 ± 7.500	91.900 ± 1.500	88.100 ± 10.600	99.000 ± 12.800
**3h**^**(a)**^	74.200 ± 1.900	86.800 ± 0.600	87.200 ± 13.900	104.500 ± 12.400
**3i**	86.000 ± 12.100	74.000 ± 1.900	78.900 ± 2.600	63.700 ± 3.800
**3j**	84.200 ± 11.300	73.600 ± 15.400	113.900 ± 44.500	94.800 ± 4.000
**3k**	–	–	107.300 ± 29.400	66.800 ± 5.500
**3l**	81.300 ± 4.500	82.900 ± 8.300	68.300 ± 13.500	81.600 ± 2.300
**3m**	83.100 ± 11.800	52.700 ± 6.100	76.200 ± 8.600	61.300 ± 7.200

^(a)^The drug concentration was 1 mM.

#### In vitro pancreatic cancer cells inhibitory activities

MTT method was used to investigate the inhibitory effect of the catalpol and its derivatives on the cell formation of PANC-1, BxPC-3 and HPDE6-C7 cells cultured in vitro. The drug concentration was 1 mg/mL. From the preliminary results in Table 3. Catalpol and its derivatives possessed relatively weak inhibitory activity on two pancreatic cancer cell lines. It can be seen that catalpol has weak cell inhibition effect. At 72 h, for the PANC-1 cell, compounds **3g** and **3k** exhibited stronger inhibitory effect than catalpol, and for the BxPC-3 cell line, compounds **3d** and **3k** exhibited stronger inhibition than catalpol. The results showed that catalpol and its derivatives treatment had almost no effect on human normal pancreatic ductal epithelial cell (HPDE6-C7). Therefore, catalpol and its derivatives might not be a significantly toxic.

**Table 3:** In vitro pancreatie cancer cells inhibitory activities of pyrazole modified catalpol derivatives.

Number of compounds	Pancreatic cancer cells viability (% of control)								
	PANC-1			HPDE6-C7			BxPC-3		
	24 h	48 h	72 h	24 h	48 h	72 h	24 h	48 h	72 h
**1**	100.345 ± 2.144	100.403 ± 0.806	99.645 ± 3.078	100.888 ± 4.893	95.986 ± 5.440	99.109 ± 4.600	98.824 ± 4.379	102.352 ± 5.031	94.519 ± 2.832
**3a**	135.484 ± 13.127	110.289 ± 7.557	112.738 ± 2.374	91.818 ± 6.121	97.223 ± 3.053	85.599 ± 2.019	117.500 ± 3.267	131.392 ± 6.959	134.325 ± 3.593
**3b**	88.966 ± 10.292	82.886 ± 4.120	109.830 ± 5.611	106.925 ± 5.035	100.258 ± 8.430	104.862 ± 1.417	63.472 ± 2.105	95.147 ± 4.373	112.142 ± 10.501
**3c**	96.853 ± 2.098	98.781 ± 11.750	102.189 ± 4.710	92.139 ± 2.162	96.757 ± 7.513	95.415 ± 4.985	117.355 ± 5.963	102.559 ± 2.167	101.207 ± 2.637
**3d**	112.500 ± 12.429	86.450 ± 5.341	121.332 ± 9.925	108.538 ± 5.179	104.720 ± 4.923	110.300 ± 4.519	83.034 ± 8.208	76.316 ± 10.747	118.874 ± 14.427
**3e**	92.573 ± 8.038	83.442 ± 7.059	119.368 ± 12.635	98.404 ± 1.321	109.785 ± 13.215	109.954 ± 5.082	69.720 ± 10.783	91.240 ± 10.271	110.862 ± 15.523
**3f**	102.972 ± 6.107	114.024 ± 6.698	98.738 ± 2.978	94.846 ± 3.341	101.978 ± 5.574	102.413 ± 2.892	123.423 ± 3.531	105.044 ± 4.558	101.552 ± 1.101
**3g**	87.390 ± 5.288	86.392 ± 7.904	111.740 ± 10.041	99.154 ± 3.688	92.500 ± 7.509	103.335 ± 1.860	86.170 ± 6.973	85.318 ± 8.100	127.670 ± 9.707
**3h**	133.871 ± 11.843	96.420 ± 8.636	113.865 ± 13.851	78.637 ± 0.580	84.895 ± 6.675	89.762 ± 6.158	102.500 ± 5.862	118.052 ± 10.256	151.275 ± 14.853
**3i**	116.613 ± 22.320	125.096 ± 7.472	137.522 ± 9.674	94.621 ± 0.971	108.021 ± 3.653	106.985 ± 2.430	88.864 ± 3.511	117.734 ± 4.654	138.403 ± 2.102
**3j**	95.345 ± 5.556	99.059 ± 3.760	101.773 ± 3.132	99.873 ± 13.420	97.817 ± 5.046	95.811 ± 5.692	94.412 ± 2.549	94.221 ± 2.780	95.748 ± 1.599
**3k**	94.747 ± 5.444	79.844 ± 5.779	124.616 ± 14.725	108.628 ± 13.429	103.980 ± 10.272	83.688 ± 9.203	74.055 ± 0.445	87.627 ± 4.840	103.687 ± 7.841
**3l**	129.839 ± 12.971	98.088 ± 6.845	115.858 ± 5.335	88.712 ± 4.302	92.036 ± 8.995	90.087 ± 4.508	105.682 ± 14.685	123.134 ± 2.745	137.128 ± 4.099
**3m**	137.258 ± 8.549	92.145 ± 7.746	124.784 ± 12.678	86.591 ± 3.939	89.443 ± 10.476	91.006 ± 2.785	99.546 ± 7.216	124.246 ± 13.609	135.854 ± 4.255

### SAR for cancer cells

#### SAR for esophageal cancer cells inhibitory

**Figure 7:**
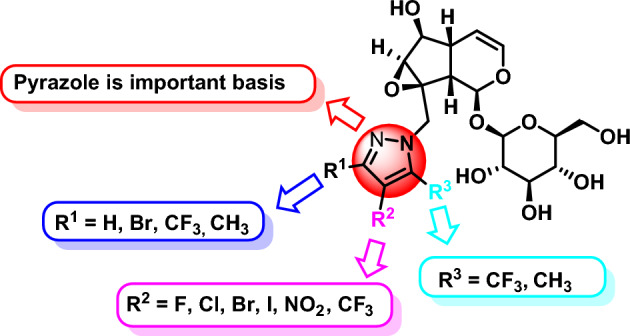
Structure–activity relationship of catalpol and its derivatives on pancreatic and esophageal cancer cells.

According to the anti-esophageal cancer data of catalpol and its derivatives, the results revealed that the parazole structures play an important role in the structure–activity relationship (SAR) for esophageal cancer cells inhibitory activities. The SAR was discussed based on variable of “R” groups (Fig. 7). The variation in the activities rely upon variable features at R^1^, R^2^ and R^3^, among them, compound **3e** with C3-position bromo (halogen) substituents of pyrazole at the C10-position of catalpol displayed the most activity. Secondly, the anti-esophageal cancer activity of R^1^ halogen substituted compounds was superior to other substituted compounds.

#### SAR for pancreatic cancer cells inhibitory

According to the anti-pancreatic cancer data of catalpol and its derivatives, the results revealed that the pyrazole derivatives with substituents structures play an important role in the structure–activity relationship (SAR) for pancreatic cancer cells inhibitory activities. The SAR was discussed based on variable of “R” groups (Fig. 7). The variation in the activities rely upon variable features at R^1^, R^2^ and R^3^, among them, catalpol and its derivatives have a weak inhibitory effect on pancreatic cancer. The activity of catalpol derivatives with substituents on pancreatic cancer is slightly stronger than that of catalpol itself.

### Mechanism

In order to study the synthesis mechanism and biological activity mechanism of catalpol, we made a preliminary exploration of computer-aided theoretical calculation.

#### Synthesis mechanism

**Figure 8:**
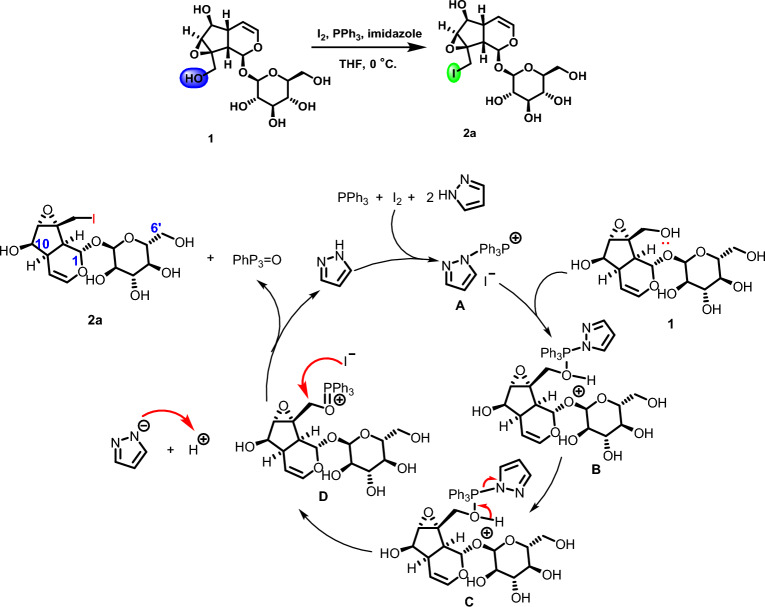
Iodination reaction mechanism.

The structure of catalpol contains six hydroxyl groups, of which two primary hydroxyl groups (C10-OH, C6′-OH) have different reactivity based on theoretical calculation, and the aglycone (C10-OH) is better than that of primary hydroxyl group on glucose (C6′-OH). According to the classical hydroxyl iodination reaction process^21^, we speculated the reaction mechanism of iodine-modified catalpol derivative was shown in Fig. 8. Imidazole, triphenylphosphine and iodine form intermediate **A**, which reacted with the catalpol to form the intermediate **B**, then N–P and O–H bonds were broken in the intramolecular to get intermediate **C**, then was followed by intermolecular nucleophilic substitution to obtain the final compound **2a**.

#### Molecular binding studies

The mechanism of pharmacological activity in this study was preliminarily explored through molecular docking. AutoDock Tools 1.5.6 was used for molecular docking in this study. It has been confirmed that the expression of VEGFR-2 in cancer tissue is closely related to tumor angiogenesis, regulation, occurrence, development and metastasis of cancer ^22^. According to the previous literature research, it was found that VEGFR-2 was highly expressed in pancreatic cancer and esophageal cancer^23,24^. We selected a crystallographic structure of human VEGFR-2 protein tyrosine kinase (PDB ID: 4AGD) as a potential target, which is extracted from the RCSB PDB (RCSB Protein Data Bank) database. The obtained 13 compounds **3a–3m** and the original protein ligand (ligand) were pretreated and stored in pdbqt format after structural optimization and charge calculation. The protein crystal was pretreated by deliberately removing water molecules and non-standard residues and replacing them with hydrogen atoms. The protein was set to be rigid, the small molecules were set to be flexible, and the conformation search was performed using Lamarck genetic algorithm, the center coordinate of the lattice box is x = 51.335 y = − 2.78 z = − 15.563, and the number of runs is 10.

**Figure 9:**
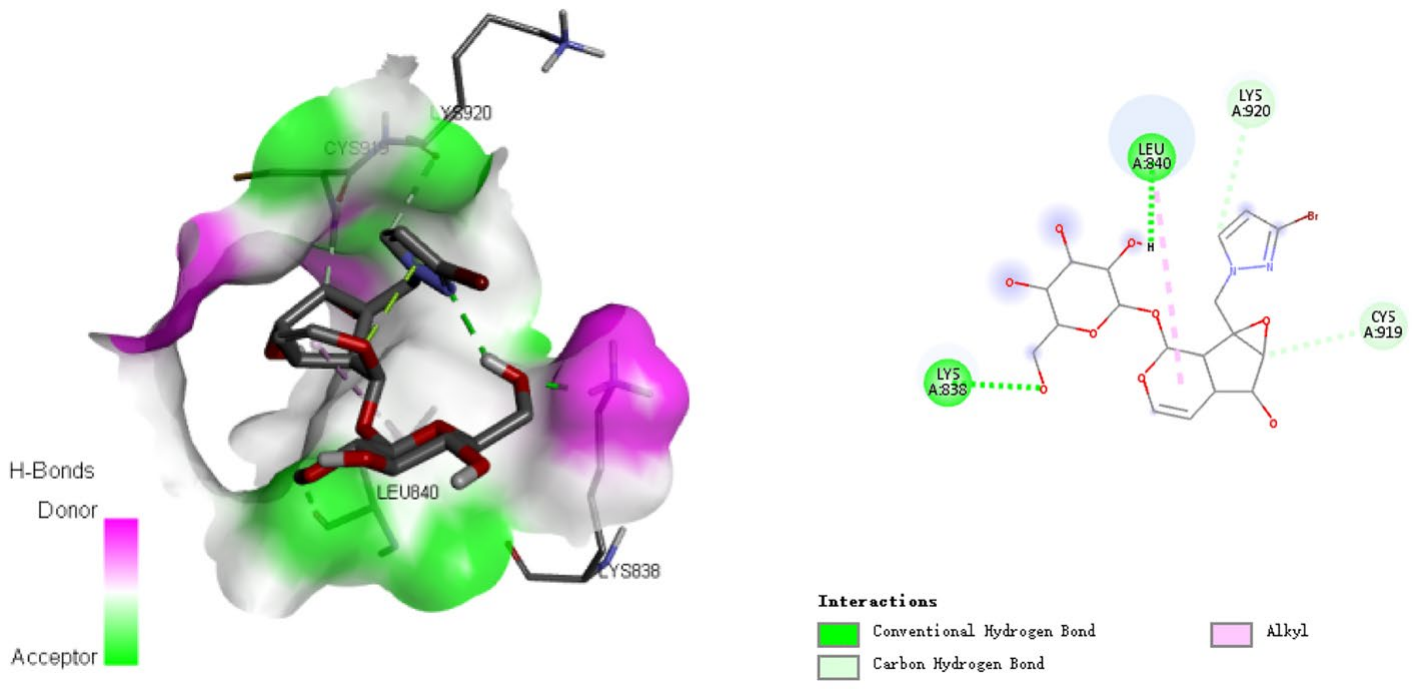
The interactions between compound **3e** and VEGFR-2.

**Figure 10:**
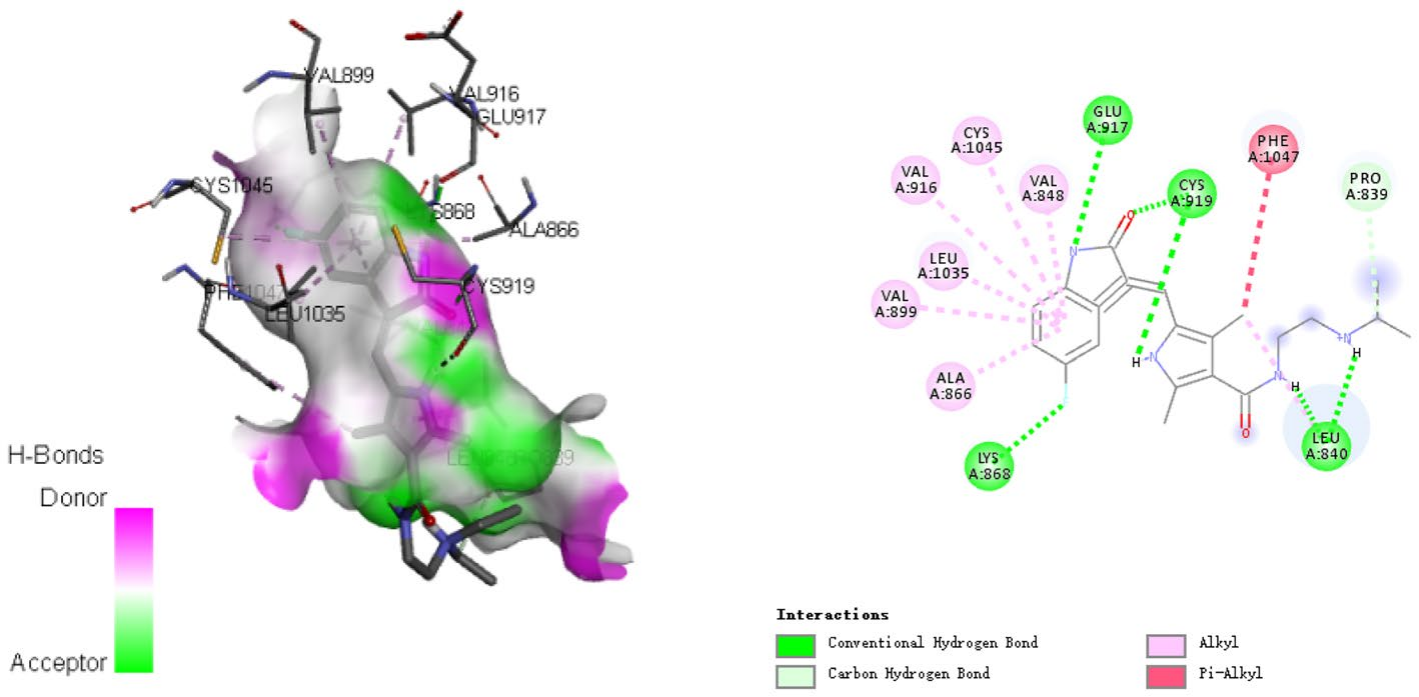
The interactions between ligand and VEGFR-2.

The 13 compounds were connected with ligand obtained with the VEGFR-2 binding pocket. In the analysis of protein docking score, it was found that the synthesized pyrazole modified catalpol derivatives and VEGFR-2 could be docked well. The specific docking score results are shown in Table 4. By docking scoring, the best compounds **3e** were selected to bind to the receptor protein and their interaction with the target enzyme was investigated. The free energy of binding of compound **3e** to the target enzyme was found to be − 6.97 kcal/mol, which was significantly higher than that of catalpol (− 4.5 kcal/mo1). This demonstrates that the catalpol derivatives designed for synthesis all have strong target enzyme interactions. Using the scoring function in Discovery Studio 2016 Client, analysis of the binding pattern with the target enzymes showed that there are four main interactions between ligand and protein, namely hydrophobic binding alkyl interaction, hydrocarbon bond, hydrogen bond interaction, and π-alkyl interaction. Compound **3e** mainly interacts with amino acid residues LEU840 to form hydrophobic binding alkyl, and forms hydrocarbon bonds with amino acid residues LYS920 and CYS919, while the hydroxyl group on the parent nucleus of catalpol derivatives forms hydrogen bonds with amino acid residues LEU840 and LYS838, which increases the combination of compound molecules and target enzymes. It can be seen that the binding action of compound **3e** with 4AGD and the binding action mode of protein with the original ligand are similar. It can be speculated that compound **3e** has potential inhibitory effect on VEGFR-2. The interactions were shown in Figs. 9 and 10.

**Table 4:** Scores of docking between target compounds and related proteins.

Human epidermal growth factor receptor (PDB ID: 4AGD)	
Compound	Binding free energy (kcal/mol)
3a	− 5.71
3b	− 5.76
3c	− 6.63
3d	− 4.48
3e	− 6.97
3f	− 5.14
3g	− 5.45
3h	− 5.42
3i	− 5.49
3j	− 6.13
3k	− 4.18
3l	− 5.38
3m	− 4.58
Ligand	− 8.8

## Conclusion

Catalpol is a polyhydroxy compound and it is challenging to modify its hydroxyl at the specific position. In this paper, a simple method for pyrazole heterocyclic modification at C10-position hydroxyl of catalpol was invented without damaging the parent of catalpol and a series of catalpol derivatives were synthesized by using the principle of drug combination. The anti-esophageal activity and anti-pancreatic cancer activity of these pyrazole modified catalpol derivatives were tested. The reaction mechanism and activity mechanism were preliminarily explored. Finally, the compound **3e** has good anti-esophageal activity and is expected to develop into a new candidate drug.

## Supplementary Information

Supplementary Information.

## Data Availability

All data generated or analysed for this study are included in this published paper (and its Supplementary Information files).
