# Supplementary material for: Design, synthesis and anticancer activities evaluation of novel pyrazole modified catalpol derivatives
Source: Sci Rep. 2023 May 12;13:7756. doi: 10.1038/s41598-023-33403-9 (PMC10182059; doi:10.1038/s41598-023-33403-9)
Supplement: Supplementary file 1 — Supplementary Information. [file 41598_2023_33403_MOESM1_ESM.docx]

**Design, Synthesis and Anticancer Activities Evaluation of** **Novel Pyrazole Modified Catalpol Derivatives**

Yuanfang Kong ^a,1^, Shuanglin Liu ^a,b,c,1^, Shaopei Wang ^a,b,c^, Bin Yang ^a^, Wei He ^a^, Hehe Li ^a^, Siqi Yang ^a^, Guoqing Wang ^d,^ * and Chunhong Dong ^a,b,c, *^

^a^ *Henan University of Chinese Medicine, Zhengzhou 450046, Henan, China.*

*^b^ Henan Polysaccharide Research Center, Zhengzhou, 450046, Henan, China.*

*^c^ Henan Key Laboratory of Chinese Medicine for Polysaccharides and Drugs Research, Zhengzhou, 450046, Henan, China.*

*^d^ Department of Applied Chemistry, Zhengzhou University of Light Industry, Zhengzhou 450001, Henan, China.*

^1^*These authors contributed equally: Yuanfang Kong and Shuanglin Liu*

*Correspondence author: E-mail: [gqwang@zzuli.edu.cn](mailto:gqwang@zzuli.edu.cn), [chunhong_dong@hactcm.edu.cn](mailto:chunhong_dong@hactcm.edu.cn)

**Experimental**

*1. Reagents and instruments*

All reagents and solvents were obtained from commercial sources and used without further purification unless otherwise indicated. All reactions were performed in oven-dried glass ware and were monitored for completeness by thin-layer chromatography (TLC) using silica gel (visualized by UV light or developed by treatment with anisaldehyde stain or iodine stain). All the chemical structures are drawn using ChemDraw 15.1.lnk software. ^1^H NMR and ^13^C NMR spectra were recorded on a Bruker AV-400 spectrometer at 500 MHz and 125 MHz, or Bruker AV-600 spectrometer at 600 and 150 MHz, respectively, in DMSO-*d_6_*, using DMSO-*d_6_* as the reference standard (2.50 ppm). Chemical shifts are reported in ppm (δ) relative to tetramethylsilane (TMS) as an internal standard. Multiplicities were given as s (singlet), brs (broad singlet), d (doublet), t (triplet), q (quartet), p (pentet) and m (multiplet). Coupling constants (*J*) are reported in Hz. All the experiments were recorded and data were processed using standard Bruker software. MS data were obtained on a Mainer System Saimofei LCQ fleet mass spectrometer. Thin-layer chromatography was performed on silica gel 60 F_254_ (Qingdao Marine Chemical Ltd., P. R. China). Column chromatography purification was conducted on silica gel (200–300 mesh, Qingdao Marine Chemical Ltd., P. R. China).

**2. Synthesis**

*2.1. Synthesis of intermediate* ***2a***

To a stirring solution of catalpol (36.2 mg, 0.1 mmol) in 1 mL ultra-dry THF, imidazole (85.7 mg, 1.26 mmol), triphenylphosphine (157.4 mg, 0.6 mmol) and iodine (152.3 mg, 0.6 mmol) was added in the presence of nitrogen atmosphere at 0 ^o^C. Catalpol disappeared in the reaction mixture after 18 h by TLC monitored, and then methanol was added, the solvent was evaporated and the crude product purified by silica gel column chromatography with dichloromethane and methanol (*v*/*v* = 15:1) to produce intermediate **2a**.

Compound **2a**, yield, 70 %; **^1^H NMR** (500 MHz, DMSO-*d*_6_) δ 6.37 (dd, *J* = 5.9, 1.8 Hz, 1H), 5.33 (s, 1H), 5.05 (s, 1H), 4.98 (dd, *J* = 6.0, 4.6 Hz, 2H), 4.89 (d, *J* = 9.7 Hz, 1H), 4.58 (d, *J* = 7.4 Hz, 1H), 4.11 (d, *J* = 10.4 Hz, 1H), 3.81 – 3.75 (m, 1H), 3.68 (dd, *J* = 11.9, 2.0 Hz, 1H), 3.66 – 3.62 (m, 1H), 3.38 (dd, *J* = 11.8, 6.8 Hz, 1H), 3.23 (d, *J* = 10.4 Hz, 1H), 3.21 – 3.10 (m, 5H), 3.03 – 2.95 (m, 1H), 2.36 (dd, *J* = 9.7, 7.6 Hz, 1H), 2.12 (m, *J* = 8.0, 4.6, 1.8 Hz, 1H). **^13^C NMR** (125 MHz, DMSO-*d*_6_) δ 140.80, 103.28, 99.18, 94.33, 78.28, 77.74, 77.19, 73.75, 70.58, 68.89, 63.58, 61.84, 49.07, 42.75, 37.48. **HRMS** (ESI+) Calculated for C_15_H_25_NIO_9_ [M+NH_4_] ^+^: 490.0562, found: 490.0565.

*2.2. General synthesis of compounds* ***3a-3m***

Intermediate **2a** (1 mmol) was dissolved in DMF solvent, and the substituted imidazoles, or substituted pyrazoles (6.6 mmol) and anhydrous potassium carbonate (2.2 mmol) was added into under stirring at room temperature. Then the mixture heated to 70 ^o^C for reaction until the intermediate **2a** disappeared by TLC monitor. Finally, methanol was added into the reaction mixture after cooling, and the solvent was evaporated, the crude product purified by silica gel column chromatography with dichloromethane and methanol from 20: 1 to 3: 1 to obtain compounds **3a-3m.**

Compound **3a**, yield, 70 %; **^1^H NMR** (500 MHz, DMSO-*d*_6_) δ 7.68 (d, *J* = 2.3 Hz, 1H), 7.41 (d, *J* = 1.9 Hz, 1H), 6.40 (d, *J* = 6.0 Hz, 1H), 5.27 (d, *J* = 5.4 Hz, 1H), 5.18 (d, *J* = 5.1 Hz, 1H), 5.01 (m, *J* = 15.0, 5.1 Hz, 4H), 4.93 (d, *J* = 15.2 Hz, 1H), 4.76 (m, *J* = 6.6, 2.9 Hz, 1H), 4.70 (d, *J* = 15.2 Hz, 1H), 4.66 (d, *J* = 7.9 Hz, 1H), 3.78 (dd, *J* = 8.2, 5.6 Hz, 1H), 3.77 – 3.70 (m, 1H), 3.48 (dt, *J* = 12.0, 6.0 Hz, 2H), 3.20 (m, *J* = 9.7, 5.0 Hz, 3H), 3.14 – 3.06 (m, 2H), 2.35 (dd, *J* = 9.8, 7.4 Hz, 1H), 2.12 (q, *J* = 7.4 Hz, 1H).**^13^C NMR** (126 MHz, DMSO-*d*_6_) δ 140.97, 138.86, 131.76, 105.72, 103.30, 99.97, 95.01, 77.51, 77.28, 76.96, 73.78, 70.27, 63.48, 61.95, 61.49, 50.34, 42.77, 37.87. **HRMS** (ESI+) Calculated for C_18_H_25_N_2_O_9_ [M+H]^+^: 413.1554, found: 413.1556.

Compound **3b**, yield, 78 %; **^1^H NMR** (500 MHz, DMSO-*d*_6_) δ 7.50 (d, *J* = 2.1 Hz, 1H), 6.38 (dd, *J* = 5.9, 1.8 Hz, 1H), 6.00 (d, *J* = 2.2 Hz, 1H), 5.23 (d, *J* = 5.4 Hz, 1H), 5.17 (d, *J* = 5.2 Hz, 1H), 5.04 – 4.97 (m, 5H), 4.84 (t, *J* = 6.7 Hz, 1H), 4.65 (dd, *J* = 7.9, 1.6 Hz, 1H), 4.52 (d, *J* = 15.4 Hz, 1H), 3.78 – 3.71 (m, 2H), 3.50 (m, *J* = 12.1, 6.2 Hz, 1H), 3.24 – 3.15 (m, 3H), 3.15 – 3.06 (m, 2H), 2.33 (dd, *J* = 9.8, 7.4 Hz, 1H), 2.13 (s, 4H). **^13^C NMR** (151 MHz, DMSO-*d*_6_) δ 147.35, 141.01, 132.58, 105.27, 103.27, 100.23, 95.25, 77.37, 77.29, 76.93, 73.79, 70.13, 63.67, 61.80, 61.30, 49.79, 42.76, 37.92, 13.53. **HRMS** (ESI+) Calculated for C_19_H_27_N_2_O_9_ [M+H]^+^: 427.1711, found: 427.1711.

Compound **3c**, yield, 55 %;**^1^H NMR** (500 MHz, DMSO-*d*_6_) δ 7.49 (d, *J* = 2.2 Hz, 1H), 6.38 (dd, *J* = 6.0, 1.8 Hz, 1H), 6.00 (d, *J* = 2.2 Hz, 1H), 5.24 – 5.16 (m, 2H), 5.04 – 4.97 (m, 5H), 4.96 (s, 1H), 4.84 (q, *J* = 6.3, 5.8 Hz, 1H), 4.64 (d, *J* = 7.8 Hz, 1H), 4.52 (d, *J* = 15.4 Hz, 1H), 3.75 (m, *J* = 12.4, 10.5, 6.0 Hz, 2H), 3.49 (m, *J* = 11.6, 5.6 Hz, 1H), 3.19 (m, *J* = 7.9, 2.2 Hz, 2H), 3.13 – 3.06 (m, 2H), 2.70 (s, 1H), 2.33 (dd, *J* = 9.8, 7.4 Hz, 1H), 2.12 (s, 3H).**^13^C NMR** (151 MHz, DMSO-*d*_6_) δ 147.35, 141.01, 132.58, 105.27, 103.27, 100.22, 95.24, 77.36, 77.28, 76.92, 73.79, 70.13, 63.66, 61.81, 61.30, 49.80, 42.76, 37.91, 13.54. **HRMS** (ESI+) Calculated for C_19_H_27_N_2_O_9_ [M+H]^+^: 427.1711, found: 427.1711.

Compound **3d**, yield, 83 %; **^1^H NMR** (500 MHz, DMSO-*d*_6_) δ 6.39 (dd, *J* = 5.9, 1.9 Hz, 1H), 5.24 (s, 1H), 5.21 (s, 1H), 5.16 (d, *J* = 7.8 Hz, 1H), 5.01 – 4.93 (m, 3H), 4.65 (d, *J* = 7.9 Hz, 1H), 4.31 (d, *J* = 15.9 Hz, 1H), 3.79 – 3.70 (m, 2H), 3.49 (dd, *J* = 12.5, 5.9 Hz, 1H), 3.20 (m, *J* = 8.2, 2.6 Hz, 2H), 3.13 – 3.06 (m, 2H), 2.52 (s, 3H), 2.46 (s, 1H), 2.40 (dd, *J* = 9.8, 7.4 Hz, 1H), 2.35 (s, 1H), 2.16 (s, 3H), 2.06 (s, 3H).**^13^C NMR** (126 MHz, DMSO-*d*_6_) δ 146.73, 141.39, 141.08, 104.94, 103.27, 100.44, 95.48, 77.34, 77.21, 76.97, 73.80, 70.17, 64.50, 61.25, 61.14, 46.25, 43.18, 37.97, 13.45, 11.34. **HRMS** (ESI+) Calculated for C_19_H_27_N_2_O_9_ [M+H]^+^: 441.1867, found: 441.1867.

Compound **3e**, yield, 68 %; **^1^H NMR** (500 MHz, DMSO-*d*_6_) δ 7.73 (d, *J* = 2.3 Hz, 1H), 6.42 – 6.37 (m, 2H), 5.30 (d, *J* = 5.5 Hz, 1H), 5.15 (d, *J* = 5.2 Hz, 1H), 5.02 (dd, *J* = 10.5, 5.5 Hz, 4H), 4.79 (d, *J* = 15.3 Hz, 1H), 4.66 – 4.60 (m, 2H), 4.56 – 4.50 (m, 1H), 3.83 (dd, *J* = 8.4, 5.4 Hz, 1H), 3.73 (m, *J* = 11.9, 7.0, 2.1 Hz, 1H), 3.48 (m, *J* = 11.8, 5.7 Hz, 1H), 3.20 (m, *J* = 9.8, 6.4, 2.6 Hz, 2H), 3.11 – 3.05 (m, 3H), 2.33 (dd, *J* = 9.7, 7.5 Hz, 1H), 2.13 (m, *J* = 9.6, 7.6, 4.6, 1.8 Hz, 1H).**^13^C NMR** (151 MHz, DMSO-*d*_6_) δ 140.91, 134.59, 124.42, 108.47, 103.33, 99.66, 94.66, 77.71, 77.24, 76.94, 73.78, 70.35, 62.86, 62.15, 61.63, 51.49, 42.70, 37.80. **HRMS** (ESI+) Calculated for C_18_H_24_BrN_2_O_9_ [M+H]^+^: 491.0659, found: 491.0655.

Compound **3f**, yield, 72 %; **^1^H NMR** (500 MHz, DMSO-*d*_6_) δ 7.95 – 7.74 (m, 1H), 7.57 – 7.38 (m, 1H), 6.39 (dd, *J* = 5.9, 1.7 Hz, 1H), 5.30 (d, *J* = 5.5 Hz, 1H), 5.17 (dd, *J* = 10.6, 5.1 Hz, 1H), 5.01 (m, *J* = 9.9, 6.6, 4.3 Hz, 4H), 4.76 – 4.60 (m, 3H), 4.57 (q, *J* = 6.2 Hz, 1H), 3.81 (dd, *J* = 8.6, 5.3 Hz, 1H), 3.73 (m, *J* = 12.0, 7.0, 2.1 Hz, 1H), 3.47 (dt, *J* = 11.8, 5.8 Hz, 1H), 3.20 (m, *J* = 6.5, 3.6 Hz, 2H), 3.08 (m, *J* = 9.3, 5.3 Hz, 2H), 2.99 (d, *J* = 5.0 Hz, 1H), 2.35 (dd, *J* = 9.7, 7.5 Hz, 1H), 2.16 – 2.08 (m, 1H).**^13^C NMR** (126 MHz, DMSO-*d*_6_) δ 140.94, 131.79, 125.91, 117.96, 103.31, 99.72, 94.72, 77.63, 77.24, 76.94, 73.77, 70.32, 63.03, 62.15, 61.58, 51.99, 42.65, 37.82. **HRMS** (ESI+) Calculated for C_18_H_24_FN_2_O_9_ [M+H]^+^: 431.1460, found: 431.1462.

Compound **3g**, yield, 65 %; **^1^H NMR** (500 MHz, DMSO-*d*_6_) δ 7.89 (s, 1H), 7.51 (s, 1H), 6.38 (dd, *J* = 5.9, 1.8 Hz, 1H), 5.29 (s, 1H), 5.15 (s, 1H), 5.00 (dt, *J* = 10.6, 4.0 Hz, 4H), 4.76 (d, *J* = 15.2 Hz, 1H), 4.70 – 4.61 (m, 2H), 4.55 (q, *J* = 13.6, 12.1 Hz, 1H), 3.81 (d, *J* = 8.2 Hz, 1H), 3.72 (d, *J* = 11.8 Hz, 1H), 3.46 (dd, *J* = 11.9, 6.1 Hz, 1H), 3.22 – 3.17 (m, 2H), 3.06 (m, *J* = 8.9, 5.1 Hz, 2H), 2.99 (d, *J* = 1.1 Hz, 1H), 2.34 (dd, *J* = 9.7, 7.5 Hz, 1H), 2.11 (m, *J* = 7.8, 4.6, 1.7 Hz, 1H).**^13^C NMR** (151 MHz, DMSO-*d*_6_) δ 140.94, 137.26, 129.75, 108.50, 103.31, 99.77, 94.74, 77.64, 77.23, 76.94, 73.77, 70.31, 62.94, 62.13, 61.59, 51.66, 42.68, 37.82. **HRMS** (ESI+) Calculated for C_18_H_24_ClN_2_O_9_ [M+H]^+^: 447.1164, found: 447.1167.

Compound **3h**, yield, 56 %; **^1^H NMR** (500 MHz, DMSO-*d*_6_) δ 7.89 (s, 1H), 7.52 (s, 1H), 6.39 (d, J = 6.1 Hz, 1H), 5.29 (s, 1H), 5.15 (s, 1H), 5.09 – 4.93 (m, 4H), 4.79 (d, J = 15.2 Hz, 1H), 4.70 (d, J = 15.2 Hz, 1H), 4.64 (d, J = 7.8 Hz, 1H), 4.55 (d, J = 6.5 Hz, 1H), 3.83 – 3.79 (m, 1H), 3.72 (d, J = 9.8 Hz, 1H), 3.47 (s, 1H), 3.24 – 3.15 (m, 2H), 3.07 (q, J = 8.3 Hz, 2H), 2.98 (s, 1H), 2.34 (m, 1H), 2.11 (m, 1H).**^13^C NMR** (126 MHz, DMSO-*d*_6_) δ 140.93, 139.33, 131.78, 103.30, 99.78, 94.76, 92.20, 77.62, 77.21, 76.94, 73.77, 70.30, 62.96, 62.11, 61.58, 51.54, 42.70, 37.81. **HRMS** (ESI+) Calculated for C_18_H_24_BrN_2_O_9_ [M+H]^+^: 491.0659, found: 491.0656.

Compound **3i**, yield, 75 %; **^1^H NMR** (500 MHz, DMSO-*d*_6_) δ 7.84 (s, 1H), 7.50 (s, 1H), 6.39 (dd, *J* = 6.0, 1.7 Hz, 1H), 5.29 (d, *J* = 5.5 Hz, 1H), 5.15 (d, *J* = 5.2 Hz, 1H), 5.04 – 4.98 (m, 4H), 4.83 – 4.72 (m, 2H), 4.64 (d, *J* = 7.8 Hz, 1H), 4.57 (t, *J* = 6.3 Hz, 1H), 3.81 (dd, *J* = 8.3, 5.4 Hz, 1H), 3.73 (m, *J* = 12.1, 7.0, 2.2 Hz, 1H), 3.47 (m, *J* = 11.7, 5.8 Hz, 1H), 3.20 (m, *J* = 9.4, 6.0, 3.6 Hz, 2H), 3.07 (m, *J* = 13.8, 9.0, 5.2 Hz, 2H), 2.94 (s, 1H), 2.34 (dd, *J* = 9.7, 7.5 Hz, 1H), 2.12 (m, *J* = 7.7, 4.8, 1.7 Hz, 1H).**^13^C NMR** (151 MHz, DMSO-*d*_6_) δ 143.84, 140.94, 135.92, 103.30, 99.84, 94.79, 77.63, 77.24, 76.96, 73.79, 70.30, 63.10, 62.07, 61.60, 57.44, 51.14, 42.73, 37.84. **HRMS** (ESI+) Calculated for C_18_H_24_IN_2_O_9_ [M+H]^+^: 539.0521, found: 539.0519.

Compound **3j**, yield, 83 %; **^1^H NMR** (500 MHz, DMSO-*d*_6_) δ 8.71 (s, 1H), 8.24 (s, 1H), 6.38 (dd, *J* = 5.9, 1.7 Hz, 1H), 5.34 (d, *J* = 5.6 Hz, 1H), 5.13 – 4.88 (m, 7H), 4.66 – 4.60 (m, 2H), 4.54 (t, *J* = 6.1 Hz, 1H), 3.85 (dd, *J* = 8.3, 5.2 Hz, 1H), 3.76 – 3.69 (m, 1H), 3.53 – 3.48 (m, 1H), 3.20 (dt, *J* = 9.8, 2.8 Hz, 2H), 3.11 (dd, *J* = 9.3, 3.9 Hz, 1H), 3.02 (m, *J* = 8.5, 3.8 Hz, 1H), 2.37 (dd, *J* = 9.7, 7.5 Hz, 1H), 2.13 (m, *J* = 7.6, 4.7, 1.8 Hz, 1H). **^13^C NMR** (126 MHz, DMSO-*d*_6_) δ 140.87, 135.79, 135.36, 132.00, 103.23, 99.90, 94.72, 77.66, 77.23, 76.90, 73.67, 70.17, 62.23, 61.94, 61.53, 49.06, 42.72, 37.77. **HRMS** (ESI+) Calculated for C_18_H_24_N_3_O_11_ [M+H]^+^: 458.1405, found: 458.1405.

Compound **3k**, yield, 90 %; **^1^H NMR** (500 MHz, DMSO-*d*_6_) δ 7.55 (s, 1H), 6.40 (dd, *J* = 5.9, 1.8 Hz, 1H), 5.31 (d, *J* = 5.8 Hz, 1H), 5.12 – 4.93 (m, 7H), 4.87 (d, *J* = 15.7 Hz, 1H), 4.60 (d, *J* = 7.9 Hz, 1H), 4.52 (dd, *J* = 7.9, 4.7 Hz, 1H), 3.91 – 3.85 (m, 1H), 3.72 (m, *J* = 11.9, 7.7, 2.1 Hz, 1H), 3.42 (m, *J* = 11.7, 7.1, 4.6 Hz, 1H), 3.18 (m, *J* = 7.4, 2.7 Hz, 2H), 3.04 (s, 1H), 2.98 (m, *J* = 8.1, 5.1, 3.0 Hz, 2H), 2.14 (m, *J* = 7.9, 4.5, 1.9 Hz, 1H). **^13^C NMR** (126 MHz, DMSO-*d*_6_) δ 140.97, 140.50, 133.95, 122.04, 120.51, 107.51, 103.34, 98.84, 93.74, 77.97, 77.07, 76.90, 73.75, 70.59, 61.97, 61.74, 61.69, 50.93, 43.88, 37.82. **HRMS** (ESI+) Calculated for C_20_H_23_F_6_N_2_O_9_ [M+H]^+^: 571.1122, found: 571.1121.

Compound **3l**, yield, 89 %; **^1^H NMR** (500 MHz, DMSO-*d*_6_) δ 7.97 – 7.93 (m, 1H), 6.70 (d, *J* = 2.3 Hz, 1H), 6.39 (dd, *J* = 6.1, 1.8 Hz, 1H), 5.41 – 5.23 (m, 1H), 5.16 – 4.97 (m, 4H), 4.94 (d, *J* = 15.2 Hz, 1H), 4.68 – 4.62 (m, 2H), 4.52 (s, 1H), 3.85 (d, *J* = 8.1 Hz, 1H), 3.72 (dd, *J* = 12.0, 2.1 Hz, 1H), 3.47 (dd, *J* = 11.9, 6.3 Hz, 2H), 3.20 (m, *J* = 8.8, 2.1 Hz, 2H), 3.11 (s, 1H), 3.10 – 3.03 (m, 2H), 2.35 (dd, *J* = 9.7, 7.5 Hz, 1H), 2.11 (m, *J* = 7.7, 4.7, 1.8 Hz, 1H). **^13^C NMR** (126 MHz, DMSO-*d*_6_) δ 140.88, 134.00, 120.95, 104.68, 103.32, 99.58, 94.53, 77.76, 77.20, 76.93, 73.75, 70.37, 62.61, 62.32, 61.68, 51.89, 49.06, 42.71, 37.80. **HRMS** (ESI+) Calculated for C_19_H_24_F_3_N_2_O_9_ [M+H]^+^: 481.1428, found: 481.1426.

Compound **3m**, yield, 90 %; **^1^H NMR** (500 MHz, DMSO-*d*_6_) δ 8.27 (s, 1H), 7.87 (s, 1H), 6.39 (dd, *J* = 6.0, 1.8 Hz, 1H), 5.31 (d, *J* = 5.6 Hz, 1H), 5.11 (d, *J* = 5.1 Hz, 1H), 5.01 (m, *J* = 11.2, 5.5, 4.9 Hz, 4H), 4.87 (d, *J* = 15.2 Hz, 1H), 4.75 (d, *J* = 15.2 Hz, 1H), 4.64 (d, *J* = 7.8 Hz, 1H), 4.55 (t, *J* = 6.2 Hz, 1H), 3.83 (dd, *J* = 8.2, 5.4 Hz, 1H), 3.73 (m, *J* = 11.9, 6.9, 2.0 Hz, 1H), 3.49 (m *J* = 11.5, 5.7 Hz, 1H), 3.24 – 3.17 (m, 3H), 3.07 (m, *J* = 16.2, 8.8, 4.8 Hz, 2H), 2.36 (dd, *J* = 9.7, 7.5 Hz, 1H), 2.13 (m, *J* = 7.5, 4.6, 1.8 Hz, 1H). **^13^C NMR** (126 MHz, DMSO-*d*_6_) δ 140.91, 136.76, 131.85, 112.08, 103.26, 99.86, 94.76, 77.65, 77.23, 76.95, 73.77, 70.24, 62.73, 62.02, 61.57, 51.35, 49.06, 42.78, 37.82. **HRMS** (ESI+) Calculated for C_19_H_24_F_3_N_2_O_9_ [M+H]^+^: 481.1428, found: 481.1424.

*2.3. NMR analysis of characteristic compound*

Since the similarity of all kinds of compounds, the spectrum of compound **3k** were analyzed as an example (Supplementary Fig. S1).

According to the ^13^C NMR spectra of catalpol, ^13^C NMR (125 MHz, DMSO-*d_6_*) δ 140.70 (3C), 103.81(4C), 98.25 (1´C), 93.69 (1C), 77.88 (3´C), 77.6 (15´C), 76.83 (6C), 73.86 (2´C), 70.66 (4´C), 65.25 (8C), 61.75 (6´C), 61.14 (7C), 59.41(10C), 42.57 (9C), 37.87 (5C). Compound **3k**, ^13^C NMR (125 MHz, DMSO-*d_6_*) δ 140.97 (3C), 140.50 (3´´C), 133.95 (5´´C), 122.04 (6´´C), 120.51 (7´´C), 107.51 (4´´C), 103.34 (4C), 98.84 (1´C), 93.74 (1C), 77.97 (3´C), 77.07 (5´C), 76.90 (6C), 73.75 (2´C), 70.59 (4´C), 61.97 (8C), 61.74 (6´C), 61.69 (7C), 50.93 (10C), 43.88 (9C), 37.82 (5C). From the dept 135 spectrums of catalpol and pyrazole-modified catalpol derivatives (**3k**), it can be seen that substitution reaction in the C-10 position hydroxyl of catalpol.

Supplementary Fig. S1. Dept 135 spectrum of compound **3k** and catalpol

*2.3.1. ^1^H NMR*

^1^H NMR spectrum of compound **3h** was recorded in DMSO‑*d_6_* on a Bruker AM 500 MHz instrument (Supplementary Fig. S2). The iridoid skeleton of pyrazole-catalpol derivatives has 7 protons, and the pyrazole heterocycle contains two protons. In the pyrazole heterocyclic compound, the two hydrogens are single peaks, and the chemical shift is 7.89 and 7.52. In the iridoid structure 7H is single peak, and among them, signal of proton C-3 appeared at δ_H_ 6.40 as double doublet (*J* _3,1_ = 6.0 Hz, *J* _3,4_ = 1.7 Hz), coupling with the proton of C-1 & C-4, while the proton C-9 appeared at δ_H_ 2.39 as double doublet (*J* _9,1_ = 7.5 Hz, *J* _9,5_ = 9.7 Hz), coupling with the proton of C-1 & C-5, 5H and 7H are multiplet peaks.

Supplementary Fig. S2. ^1^H NMR chemical shifts of representative compound **3h**

*3. MTT method for screening for anticancer activity*

2000-4000 cells were seeded in a 96-well plate which were adhered for 24 h, and catalpol or 10-position heterocyclic catalpols was added. After the incubator has been used for a certain period of time at 37 ^o^C, 10 μL of MTT was added to each well and placed in the incubator to continue incubating for 4 h. The medium and MTT were discarded then DMSO was added to each well. The culture plate was placed on a shaker to shake until the formazan was fully dissolved to detect the optical density (OD) of each well at a wavelength of 490 nm with a microplate reader, and the cell viability was calculated according to the following formula:

Cell survival rate = (treatment group OD - zero adjustment hole OD) / (control group OD - zero adjustment hole OD) × 100%

*3.1.* *Esophageal cancer cells inhibitory activities*

Supplementary Fig. S3. Inhibitory effect of catalpol on Eca109 and EC9706 cells

Supplementary Fig. S4. Inhibitory effect of compound **3e** on Eca109 and EC9706 cells

**Electronic Supplementary Information**

Copies of ^1^H and ^13^C NMR spectra of substrates and products

Supplementary Fig. S5-A. ^1^H NMR spectra of compound **2a**

 Supplementary Fig. S5-B. ^13^C NMR spectra of compound **2a**

Supplementary Fig. S6-A. ^1^H NMR spectra of compound **3a**

Supplementary Fig. S6-B. ^13^C NMR spectra of compound **3a**

Supplementary Fig. S7-A. ^1^H NMR spectra of compound **3b**

Supplementary Fig. S7-B. ^13^C NMR spectra of compound **3b**

Supplementary Fig. S8-A. ^1^H NMR spectra of compound **3c**

Supplementary Fig. S8-B. ^13^C NMR spectra of compound **3c**

Supplementary Fig. S9-A. ^1^H NMR spectra of compound **3d**

Supplementary Fig. S9-B. ^13^C NMR spectra of compound **3d**

Supplementary Fig. S10-A. ^1^H NMR spectra of compound **3e**

Supplementary Fig. S10-B. ^13^C NMR spectra of compound **3e**

Supplementary Fig. S11-A. ^1^H NMR spectra of compound **3f**

Supplementary Fig. S11-B. ^13^C NMR spectra of compound **3f**

Supplementary Fig. S12-A. ^1^H NMR spectra of compound **3g**

Supplementary Fig. S12-B. ^13^C NMR spectra of compound **3g**

Supplementary Fig. S13-A. ^1^H NMR spectra of compound **3h**

Supplementary Fig. S13-B. ^13^C NMR spectra of compound **3h**

Supplementary Fig. S14-A. ^1^H NMR spectra of compound **3i**

Supplementary Fig. S14-B. ^13^C NMR spectra of compound **3i**

Supplementary Fig. S15-A. ^1^H NMR spectra of compound **3j**

Supplementary Fig. S15-B. ^13^C NMR spectra of compound **3j**

Supplementary Fig. S16-A. ^1^H NMR spectra of compound **3k**

Supplementary Fig. S16-B. ^13^C NMR spectra of compound **3k**

Supplementary Fig. S17-A. ^1^H NMR spectra of compound **3l**

Supplementary Fig. S17-B. ^13^C NMR spectra of compound **3l**

Supplementary Fig. S18-A. ^1^H NMR spectra of compound **3m**

Supplementary Fig. S18-B. ^13^C NMR spectra of compound **3m**
